# Self-harm in women in midlife: rates, precipitating problems and outcomes following hospital presentations in the multicentre study of self-harm in England

**DOI:** 10.1192/bjp.2024.215

**Published:** 2025-07

**Authors:** Caroline Clements, Harriet Bickley, Keith Hawton, Galit Geulayov, Keith Waters, Jennifer Ness, Samantha Kelly, Ellen Townsend, Louis Appleby, Nav Kapur

**Affiliations:** Centre for Mental Health and Safety, Manchester Academic Health Sciences Centre, University of Manchester, Manchester, UK; Centre for Suicide Research, Department of Psychiatry, University of Oxford, Warneford Hospital, Oxford, UK; Centre for Self-harm and Suicide Prevention Research, Derbyshire Healthcare NHS Foundation Trust, Derby, UK; School of Psychology, University of Nottingham, Nottingham, UK; NIHR Greater Manchester Patient Safety Research Collaboration, University of Manchester, Manchester, UK; Mersey Care NHS Foundation Trust, Liverpool, UK

**Keywords:** Self-harm, suicide, epidemiology, women's health, midlife

## Abstract

**Background:**

Suicide in women in the UK is highest among those in midlife. Given the unique changes in biological, social and economic risk factors experienced by women in midlife, more information is needed to inform care.

**Aim:**

To investigate rates, characteristics and outcomes of self-harm in women in midlife compared to younger women and identify differences within the midlife age-group.

**Method:**

Data on women aged 40–59 years from the Multicentre Study of Self-harm in England from 2003 to 2016 were used, including mortality follow-up to 2019, collected via specialist assessments and/or emergency department records. Trends were assessed using negative binomial regression models. Comparative analysis used chi-square tests of association. Self-harm repetition and suicide mortality analyses used Cox proportional hazards models.

**Results:**

The self-harm rate in midlife women was 435 per 100 000 population and relatively stable over time (incident rate ratio (IRR) 0.99, *p* < 0.01). Midlife women reported more problems with finances, alcohol and physical and mental health. Suicide was more common in the oldest midlife women (hazard ratio 2.20, *p* < 0.01), while psychosocial assessment and psychiatric inpatient admission also increased with age.

**Conclusion:**

Addressing issues relating to finances, mental health and alcohol misuse, alongside known social and biological transitions, may help reduce self-harm in women in midlife. Alcohol use was important across midlife while physical health problems and bereavement increased with age. Despite receiving more intensive follow-up care, suicide risk in the oldest women was elevated. Awareness of these vulnerabilities may help inform clinicians’ risk formulation and safety planning.

Self-harm is an important international public health issue and a risk factor for future suicide.^1,2^ Although self-harm is more common in women than men across the lifespan, the difference in rates narrows as age increases.^3,4^ In a previous study in England, rates of self-harm for people aged 40–59 years were 449 per 100 000 population in women and 363 per 100 000 population in men.^4^ Characteristics and outcomes also differed, with indicators of poor mental health more common in women and factors relating to financial and employment difficulties more common in men.^4^ In England, the highest suicide rates in women are among those in midlife, in particular women aged 45–49 years (7.8 per 100 000 in 2021; ONS.gov.uk). High suicide rates among women in midlife are also found across international studies.^5,6^ Systematic review and meta-analytic work has shown that risk of suicide in women in midlife increased in the presence of unemployment, and/or separation or divorce.^5^ Increased alcohol use is a significant factor in increased suicide risk in women in the USA, which is of concern as recent evidence from the UK found increases in high-risk drinking in people in midlife during the COVID-19 pandemic.^7,8^

## Suicidal behaviour in women in midlife

Although there is no definitive marker of midlife (and the concept of midlife is subject to changes in response to social, cultural, health and economic changes), it is considered a period of significant life transitions that starts around 40–45 years of age and continues until 60–65 years of age.^9^ In women, midlife may bring additional challenges, coinciding with significant biological menopausal changes associated with mental and physical health problems.^10,11^ There is evidence that increased suicidality is associated with different menopausal stages, indicating a potential role for changes in hormone levels as a factor in suicidal behaviours.^12,13^ These biological changes often occur alongside important social challenges. The Seattle Midlife Women's Health Study identified social stressors affecting women in midlife, including changing family relationships, changes in work and social life and financial insecurity. The break-up of relationships with partners, caring responsibilities for children and elderly parents, the death of parents and their own health problems were particularly salient, with women in midlife often balancing co-occurring stressors against a background of limited resources.^14,15^

Given the link between self-harm and suicide and the potential increase in biological, social and economic risk factors in women in midlife, further investigation of self-harm in this group is required. However, self-harm research in this area is limited. Where information is available it is often presented in comparison with men^4^ or includes women as a broad midlife category among multiple age groups.^3,4^ The UK gender health gap (i.e. institutionalised sexism within healthcare, and poorer service and outcomes for women as a result) is one of the largest among high-income countries (manual.co/mens-health-gap), and along with increasing recognition of the impact of menopause-related physical/mental health difficulties, it is important to investigate self-harm as a significant issue for women in midlife, without reference to or comparison with self-harm or suicide in men.^16^ This study is a detailed investigation of self-harm in women in midlife examining rates, characteristics and outcomes in between-group comparisons with younger adult women, and within-group comparisons using 5-year age bands, to identify differences in support and care needs during the complex period of transition associated with midlife, and to explore risk and vulnerabilities in this group to help guide clinical and prevention services. The specific aims were as follows: (a) compare rates, key characteristics, repetition of self-harm and suicide mortality in women in midlife to those seen in younger women to identify differences specific to self-harm in midlife; (b) compare reported problems that precipitated self-harm between midlife and younger women, and identify any that may be related specifically to the experiences of women in midlife; and (c) repeat the above analysis stated in aims (a) and (b) using 5-year within-group comparisons to identify differences across the midlife period.

## Method

### The Multicentre Study of Self-harm in England cohort

The Multicentre Study of Self-harm in England collects information on general hospital emergency department presentations for self-harm in three hospitals in Manchester, one hospital in Oxford and one hospital in Derby. Basic data on self-harm (e.g. method, time and date) and individual demographic information (e.g. age, gender as specified in the medical records, place of residence) are collected for all presentations from hospital records, with further information (e.g. clinical history, self-reported problems that precipitated the self-harm, referrals for follow-up care) collected for people referred to psychiatric liaison teams for psychosocial assessment (or where a data collection form was completed by emergency department staff in Manchester). The Multicentre Study database used here includes information on all self-harm presentations across the three study sites from 1 January 2003 to 31 December 2016, with mortality follow-up information via data linkage with information from the Office for National Statistics (ONS) up to 31 December 2019.

### Women in midlife and comparison group

Midlife was defined as people aged 40–59 years to maintain consistency with previous work.^4^ The midlife cohort was any woman aged 40–59 years at their index (i.e. first) self-harm presentation recorded on the database (see data flowchart in Supplementary Fig. 1). The younger comparison group was defined as any woman aged 25–39 years at their index presentation. Within-group comparisons used 5-year age bands (i.e. 40–44, 45–49, 50–54 and 55–59 years at index presentation) to compare different stages of midlife. The Multicentre Study identifies gender as reported by the clinician and/or in the patients’ records. In general clinicians use information from existing patient records. For new patients, information held on the National Health Service (NHS) spine may be used. Records are only likely to be changed if the topic of sex and gender is raised within an assessment and the patient indicates a difference.

### Rates

Annual rates (per 100 000 of the age and gender-matched population) were calculated using the first self-harm presentation for each person within each calendar year. Denominators were annual ONS population estimates for Oxford City, City of Manchester and Derby Unitary Area, which match the catchment areas of the study hospitals.

### Mortality follow-up

All individuals on the Multicentre Study database were traced up to 31 December 2019. Follow-up ended if the data linkage indicated that person had died or emigrated outside of the UK. Cause of death was based on ICD-10^17^ codes for suicide (X60–X84) or event of undetermined intent (Y10–Y34). People were excluded if they could not be traced.

### Repetition of self-harm

Twelve-month repetition of self-harm was calculated as a repeat presentation to a hospital in the same study area, by the same person, within 12 months of their index self-harm episode. People that presented from 1 January 2003 to 31 December 2015 were included. Follow-up was for 12 months following the initial self-harm presentation. Follow-up ended at the first self-harm repetition, or at 12 months if there was no repetition. Repetition at any time during the study period was also assessed to compare longer-term risk. For this analysis follow-up ended on 31 December 2016. All people were included until the end of follow-up, or until a repeat self-harm presentation occurred.

### Ethical approval

Oxford and Derby self-harm monitoring projects have approval from local research ethics committees (Oxford: South Central Berkshire REC, 08/H0607/7; Derby: Derbyshire REC, 06/Q2401/84). Data collection in Manchester is carried out as a clinical audit in agreement with study sites and ratified by a local research ethics committee (South Manchester REC). All projects are fully compliant with the Data Protection Act of 1998 and have approval under Section 251 of the NHS Act 2006 to collect patient-identifiable information without patient consent.

### Statistical analyses

Trends in rates of self-harm were assessed using negative binomial regression models to account for overdispersion in the data. Descriptive statistics and chi-square tests of association were used for comparison of demographic, clinical and precipitating problem information. For additional context we compared key characteristics by centre. As some definitions of midlife include people aged 60–64 years, we conducted an additional comparison comparing the characteristics of women aged 60–64 years with the oldest age group included in our midlife sample (those aged 55–59 years). People were excluded pairwise where data were missing; therefore, each analysis only included people with a valid yes or no response, but people were otherwise retained within the primary data source (e.g. the denominator figure in percentage calculations may change for different variables). Twelve-month repetition, repetition at any time following the index presentation and mortality follow-up were analysed using Cox proportional hazards models. The main analyses were unadjusted but we also present subgroup analyses adjusted for some of the main variables associated with suicide mortality on the basis of previous literature (i.e. previous self-harm, current psychiatric care and alcohol at the time of the self-harm). These covariates were only available for the subgroup of our sample who had received a psychosocial assessment (approximately 60%).

## Results

Twenty-eight per cent of self-harm presentations by adult women aged 18 years and over (51 036; 25 610 individuals) during the study period were made by women aged 40–59 years (*n* = 14 412, 28.2%), made by 6441 (25.2%) individuals. The comparison group of younger adult women aged 25–39 years included 18 706 (36.7%) self-harm presentations made by 8850 (35.6%) individuals.

### Broad age-group comparisons between women in midlife and younger women

#### Rates

**Fig. 1:**
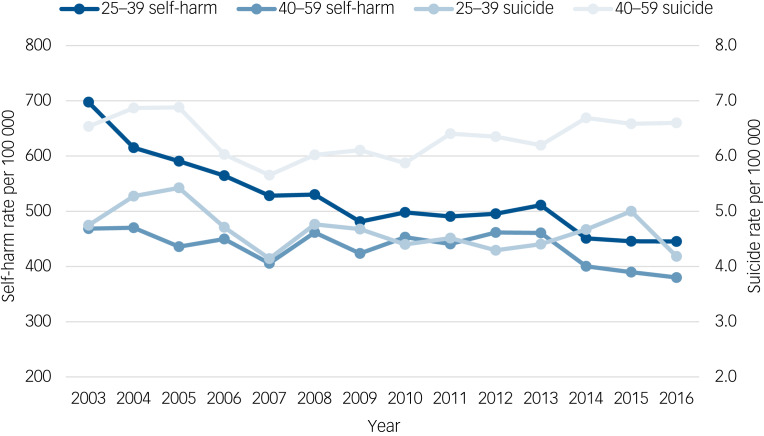
Rates of hospital presentation for self-harm and suicide rates (on secondary axis) per 100 000 of age and gender-matched populations in women aged 40–59 years and women aged 25–39 years.

Standardised rates of self-harm per 100 000 of the population were 435 for women in midlife and 520 for younger women. The incident rate ratio (IRR) showed a small decrease in annual rates of self-harm for women in midlife (IRR 0.99, 95% CI 0.98–1.00, *P* < 0.01) and younger women (IRR 0.97, 95% CI 0.96–0.98, *P* < 0.01). Rates are shown in Fig. 1 along with age and gender-matched rates of suicide in England based on figures published by the ONS.

#### Characteristics

Women in midlife more often used self-poisoning alone as the main method of self-harm compared to younger women (84.9% *v.* 79.8%; χ^2^ 66.8 (2) *P* < 0.01; see Supplementary Table 1 available at https://doi.org/10.1192/bjp.2024.215 for details), and more often received a specialist psychosocial assessment by psychiatric liaison staff, but the difference was small (62.1% *v.* 57.8%; χ^2^ 27.9 (1) *P* < 0.01).

Unemployment was similar across both groups (31.8% *v.* 31.3%), with younger women more often being employed (40.6% *v.* 43.4%). Across groups most women were White but there were more women of Black, South Asian and Other minority ethnicities in the younger age group (e.g. South Asian 2.5% *v.* 6.4%; χ^2^ 161.7 (3) *P* < 0.01). Alcohol was more often involved in the self-harm of women in midlife (64.3% *v.* 59.5%, χ^2^ 27.3 (1) *P* < 0.01), and women in midlife were more often currently receiving psychiatric care (23.7% *v.* 20.4%; χ^2^ 69.1 (1) *P* < 0.01), although these differences were small. There were no significant differences in previous self-harm.

Problems reported as precipitants of self-harm were broadly similar between groups (see Supplementary Table 1 for details). There were small but statistically significant differences seen in problems related to relationship with partner, drug problems and abuse, which were all less common in women in midlife. However, financial problems, alcohol problems, physical health problems, mental health problems and bereavement were all more common in women in midlife.

#### Characteristics by research site

Differences by research site mirrored the socioeconomic profiles of local populations. There was more deprivation, ethnic diversity and unemployment in Manchester, and women in Manchester were less likely to receive a specialist psychosocial assessment (50.0% *v.* 80.0% in Oxford and 69.5% in Derby; χ^2^ 434.8 (2) *P* < 0.01) or to be under current psychiatric care (20.9% *v.* 22.4% Oxford and 29.2% Derby; χ^2^ 28.8 (2) *P* < 0.01), but had the highest proportion of previous self-harm (59.2% *v.* 50.3% Oxford and 51.5% Derby; χ^2^28.1 (2) *P* < 0.01). In Oxford there were more problems with alcohol (29.2% *v.* 25.7% Manchester and 21.6% Derby; χ^2^ 16.9 (2) *P* < 0.01). In Derby there was a substantially higher proportion of mental health problems reported (41.2% *v.* 22.8% Oxford and 27.6% Manchester; χ^2^ 108.0 (2) *P* < 0.01).

#### Repetition and mortality

There was no difference in 12-month repetition of self-harm (hazard ratio 1.02, 95% CI 0.94–1.10, *P* = 0.67: person-years-at-risk [PYAR] 84 341) between women in midlife (1012; 16.7%) and younger women (1364; 16.4%). Adjusted analysis yielded similar findings with no significant differences between women in midlife and younger women.

Mortality information was available for 6147 women in midlife and 8133 younger women. More women in midlife died of all causes during follow-up (791; 12.9%) compared to younger women (463; 5.7%: hazard ratio 2.46, 95% CI 2.19–2.76, *P* < 0.01 PYAR 143 801). However, there was no significant difference between midlife (77; 1.2%) and younger women (83; 0.9%) in suicide mortality (hazard ratio 1.31, 95% CI 0.93–1.73, *P* = 0.13: PYAR 143 801). In adjusted analysis women in midlife were more likely to die by suicide compared to younger women (hazard ratio 1.67, 95% CI 1.08–2.57, *P* = 0.02; PYAR 78 240), with receipt of current psychiatric care also significantly associated with suicide mortality (hazard ratio 2.94, 95% CI 1.86–4.64, *P* < 0.01; PYAR 78 240).

### Within-group comparisons for women in midlife (5-year age bands)

#### Rates

**Fig. 2:**
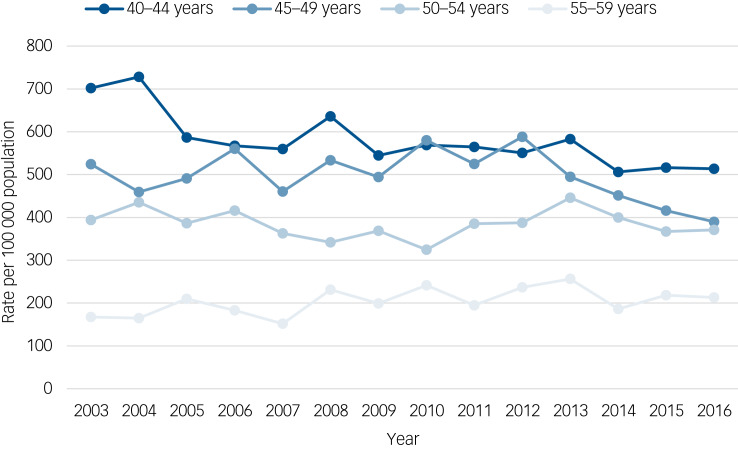
Standardised rates of hospital presentations for self-harm in women in midlife per 100 000 population within 5-year age groups.

There were 5543 (38.5%) self-harm presentations by women aged 40–44 years (2558 individuals), 4564 (31.7%) by women aged 45–49 years (1908 individuals), 2912 (20.2%) by women aged 50–54 years (1285 individuals) and 1393 (9.7%) by women aged 55–59 years (690 individuals). The overall rate of self-harm per 100 000 population decreased as the age band increased: 579.0 for 40–44 years; 497.1 for 45–49 years; 384.5 for 50–54 years; and 204.1 for women aged 55–59 years. Figure 2 shows standardised rates during the study period in women in midlife by 5-year age groups. Rates decreased over the study period in the youngest women (IRR 0.98, 95% CI 0.97–0.99, *P* < 0.01) with no significant change in women aged 45–49 years (IRR 0.99, 95% CI 0.98–1.00, *P* = 0.13) or 50–54 years (IRR 1.00, 95% CI 0.99–1.00 *P* = 0.60), and a small increase in woman aged 55–59 years (IRR 1.02, 95% CI 1.00–1.04, *P* = 0.02).

#### Characteristics

Self-harm methods were similar across midlife, with small but significant differences in specific methods of overdose. Antidepressants were less often taken in overdoses as age increased (23% to 17%) and overdoses of benzodiazepines increased with age (16% to 23%). There were no significant differences in the proportion of individuals receiving current psychiatric care, or with previous self-harm. Receipt of a specialist psychosocial assessment increased with age from 60.8% at 40–45 years to 66.1% at 55–59 years (χ^2^ 8.5 (3) *P* = 0.03).

Details of comparisons between midlife age bands are shown in Table 1. There were more South Asian women aged 40–44 years, and more women from Black and ‘Other’ ethnic groups aged 45–49 years, with ethnic diversity decreasing as age increased. The oldest group were most often unemployed and those aged 45–49 years were the least often unemployed. Indicators of deprivation (Index of Multiple Deprivation: https://data.cdrc.ac.uk/dataset/index-multiple-deprivation-imd) based on postcode of residence showed that older women were more likely to live in the most affluent areas (238; 35.9%) and the youngest women were most likely to live in the most deprived areas (1074; 44.3%; χ^2^ 11.7 (3) *P* < 0.01).

**Table 1:** Comparison of characteristics, reported problems and referrals for follow-up care among 5-year age bands of women in midlife who attended emergency departments for self-harm

Variables	40–44, *n* = 2558 (%)	45–49, *n* = 1908 (%)	50–54, *n* = 1285 (%)	55–59, *n* = 690 (%)	*X*^2^ (d.f.) *P*
All people included					
Self-poisoning	2176 (85.07)	1624 (85.12)	1084 (84.36)	584 (84.64)	4.62 (6) *P* = 0.59
Self-injury	317 (12.39)	235 (12.32)	156 (12.14)	91 (13.19)	
Self-poisoning and self-injury	65 (2.54)	49 (2.57)	45 (3.50)	15 (2.17)	
Alcohol involved	1240 (65.02)	909 (63.30)	625 (66.63)	318 (60.80)	6.06 (3) *P* = 0.109
Assessed people only					
	*n* = 1761	*n* = 1327	*n* = 904	*n* = 502	
Employment status					
Unemployed	500 (32.26)	362 (30.17)	248 (31.51)	159 (35.49)	20.32 (6) *P* = 0.002*
Employed	647 (41.74)	511 (42.58)	317 (40.28)	141 (31.47)	
Other (including student)	403 (26.00)	327 (27.25)	222 (28.21)	148 (33.04)	
Ethnicity					
White	1482 (92.11)	1154 (93.06)	789 (95.29)	448 (95.93)	20.52 (9) *P* = 0.015*
Black	34 (2.11)	28 (2.26)	10 (1.21)	5 (1.07)	
South Asian	57 (3.54)	27 (2.18)	15 (1.81)	5 (1.07)	
Other minority ethnicity	36 (2.24)	31 (2.50)	14 (1.69)	9 (1.93)	
History of self-harm	899 (57.01)	645 (54.48)	442 (54.17)	223 (50.91)	5.88 (3) *P* = 0.117
Current psychiatric care	378 (23.57)	292 (23.95)	204 (24.64)	101 (21.58)	1.62 (3) *P* = 0.65
Problems reported as precipitants of self-harm					
Relationship partner	800 (47.11)	552 (42.79)	315 (36.04)	139 (28.54)	67.33 (3) *P* < 0.001*
Relationship other family	437 (25.75)	339 (26.28)	189 (21.62)	104 (21.36)	10.11 (3) *P* = 0.018*
Relationship other	210 (12.37)	178 (13.80)	120 (13.73)	62 (12.73)	1.70 (3) *P* = 0.637
Employment	271 (15.96)	189 (14.65)	144 (16.48)	72 (14.78)	1.80 (3) *P* = 0.615
Financial	317 (18.69)	253 (19.60)	154 (17.62)	90 (18.48)	1.37 (3) *P* = 0.713
Housing	211 (12.44)	179 (13.88)	106 (12.13)	69 (14.17)	2.52 (3) *P* = 0.472
Legal	76 (4.48)	47 (3.64)	36 (4.12)	12 (2.46)	4.50 (3) *P* = 0.213
Alcohol	359 (25.14)	292 (26.24)	194 (25.97)	92 (21.55)	3.89 (3) *P* = 0.273
Drugs	54 (3.68)	39 (3.36)	23 (2.94)	9 (2.02)	3.29 (3) *P* = 0.348
Physical health	209 (12.32)	212 (16.43)	170 (19.45)	98 (20.12)	31.39 (3) *P* < 0.001*
Mental health	478 (28.13)	394 (30.52)	292 (33.37)	154 (31.62)	8.12 (3) *P* = 0.044*
Bereavement	187 (11.21)	186 (14.62)	137 (15.97)	86 (17.88)	20.02 (3) *P* < 0.001*
Abuse	199 (11.74)	148 (11.48)	79 (9.05)	43 (8.87)	6.84 (3) *P* = 0.077
Referrals from the emergency department for follow-up care					
Psychiatric outpatient	631 (36.12)	477 (36.27)	348 (39.15)	182 (36.69)	2.61 (3) 0.456
Psychiatric in-patient	82 (4.66)	73 (5.50)	58 (6.42)	42 (8.37)	11.21 (3) *P* = 0.011*
Self-discharge	23 (1.31)	24(1.81)	16 (1.77)	7 (1.39)	1.62 (3) *P* = 0.654
GP contacted	861 (53.21)	652 (53.31)	431 (51.31)	214 (46.93)	6.61 (3) 0.085
Referred to drug and alcohol services	120 (6.98)	88 (6.88)	63 (7.25)	29 (5.92)	0.92 (3) *P* = 0.820

*Indicates significance at *P* ≤ 0.05.

GP, general practitioner.

Relationship problems were the most commonly reported issues that precipitated self-harm, but the youngest women were more likely to report relationship problems with a partner as a precipitating factor (χ^2^ 67.3 (3) *P* < 0.01). Physical health problems increased with age and were most common in women aged 55–59 years (χ^2^ 31.4 (3) *P* < 0.01). Mental health problems were higher in women aged over 50 years (χ^2^ 8.1 (3) *P* = 0.04). Bereavement as a precipitating problem increased with age (χ^2^ 20.0 (3) *P* < 0.01). Referrals from the emergency department for follow-up care were similar across midlife, but older women were more often referred to in-patient psychiatric care (χ^2^ 11.2 (3) *P* = 0.01).

Additional analysis comparing women aged 55–59 years with women aged 60–64 years showed a continuation of the trends seen in the 5-year comparisons. Alcohol consumed at the time of the self-harm (58.0% *v.* 48.4%; χ^2^ 6.4 (1) *P* = 0.01), unemployment (35.5% *v.* 19.7%; χ^2^ 64.0 (1) *P* < 0.01) and financial problems (18.5% *v.* 11.2%; χ^2^ 7.6 (1) *P* < 0.01) were more common in women aged 55–59 years. Older women aged 60–64 years were more often under current psychiatric care (28.8% *v.* 21.6%; χ^2^ 5.0 (1) *P* = 0.03) and more often reported physical health problems (27.5% *v.* 20.1%; χ^2^ 5.7 *P* = 0.02).

#### Repetition of self-harm and mortality

Cox regression models for self-harm repetition and mortality are shown in Table 2. There was no significant difference in 12-month repetition of self-harm between age bands. However, repetition at any time during the study period (e.g. not limited to 12 months) was less common in women over 50 years compared to the youngest women aged 40–44 years (hazard ratio 0.75, 95% CI 0.64–0.89, *P* < 0.01: PYAR 34 228). Adjusted analysis yielded similar findings (hazard ratio 0.76, 95% CI 0.60–0.96, *P* = 0.02; PYAR 19 208) with all additional factors significant in the model.

**Table 2:** Cox regression models comparing 12-month and all-time self-harm repetition, and mortality follow-up, including mortality by suicide, among 5-year age bands of women in midlife who attended the emergency departments for self-harm

Midlife age group	*N* (%)	Hazard ratio (95% CI)	*P*-value
12-month repetition			
40–44 (*n* = 2558)	406 (15.87)	Reference group	–
45–49 (*n* = 1908)	310 (16.25)	1.05 (0.90–1.21)	0.543
50–54 (*n* = 1285)	191 (14.86)	0.95 (0.80–1.12)	0.522
55–59 (*n* = 690)	99 (14.35)	0.93 (0.74–1.15)	0.489
PYAR 34 228			
Repetition at any time (*n* and PYAR same as above)			
40–44	825 (32.25)	Reference group	–
45–49	552 (28.93)	0.93 (0.84–1.04)	0.196
50–54	339 (26.38)	0.84 (0.74–0.96)	0.009
55–59	165 (23.91)	0.76 (0.64–0.89)	0.001
All-cause mortality			
40–44 (*n* = 2432)	226 (9.29)	Reference group	–
45–49 (*n* = 1823)	196 (10.75)	1.26 (1.04–1.53)	*P* = 0.016
50–54 (*n* = 1229)	205 (16.68)	2.12 (1.76–2.57)	*P* < 0.001
55–59 (*n* = 663)	164 (24.74)	3.18 (2.60–3.89)	*P* < 0.001
PYAR 59 381			
Suicide mortality (including undetermined intent; *n* and PYAR same as above)			
40–44	22 (0.90)	Reference group	–
45–49	21 (1.15)	1.32 (0.73–2.40)	0.364
50–54	20 (1.63)	1.93 (1.05–3.54)	0.033
55–59	14 (2.11)	2.52 (1.29–4.92**)**	0.007

PYAR, person-years-at-risk.

The proportion of deaths from all causes increased as age increased, with the oldest women three times more likely than the youngest women to have died during follow-up (hazard ratio 3.18, 95% CI 2.60–3.89, *P* < 0.01: PYAR 59 381). Suicide deaths were rare, but more than twice as common among the oldest women compared to the youngest women in midlife (hazard ratio 2.23, 95% CI 1.29–4.92, *P* < 0.01: PYAR 59 381). The findings of the adjusted mortality analysis were similar with an increase in suicide risk in the oldest midlife women compared to the youngest (hazard ratio 2.83, 95% CI 1.20–6.67, *P* = 0.02; PYAR 32 688) and a significant role for receipt of current psychiatric care (hazard ratio 4.96, 95% CI 2.69–9.16, *P* < 0.01; PYAR 32 688).

## Discussion

We investigated self-harm in women in midlife and differences in characteristics associated with self-harm at different stages of midlife, using data from the Multicentre Study over a 14-year study period with mortality follow-up to the end of 2019. Rates of self-harm were lower in women in midlife (435 per 100 000) compared to younger women (520 per 100 000). The midlife cohort more often received a specialist psychosocial assessment and were more likely to be under current psychiatric care. Women in midlife more often consumed alcohol before or during self-harm and reported more alcohol and mental health problems as precipitating factors. In younger women, relationship issues and higher levels of deprivation were common. Factors potentially related to increasing age were evident in the midlife women with more reports of physical health problems and bereavement as problems associated with self-harm. No differences were found in repetition of self-harm or suicide mortality.

Within-group comparisons largely reflected differences found in the broader comparison, with women under 50 years more similar to the younger comparison group than to the women over 50 years. Rate of self-harm decreased as age increased. Younger women in midlife were more ethnically diverse and less likely to receive a specialist psychosocial assessment in the emergency department. Older women in midlife reported more mental health problems and were more often referred to psychiatric in-patient care. There was no within-group difference in alcohol consumption at the time of the self-harm, or in alcohol as a precipitating problem. There was no difference in self-harm repetition at 12 months. However, suicide mortality was twice as common in the oldest midlife age band compared to the youngest.

The doubling of suicide risk between the youngest and oldest women in midlife is clinically important, especially in light of the increased intensity of clinical care for the older women. This suggests care needs are not being met via current psychiatric care, receipt of psychosocial assessment or psychiatric in-patient admission. Our results align with previous work on the importance of social factors in the well-being of women in midlife. Financial insecurity, health problems and bereavement were all more common in women in midlife, and problems in family relationships more common in the oldest women, the latter possibly contributing to the increased suicide risk in the oldest women in midlife.^14,15^ Addressing social stressors and challenges faced by women in midlife alongside mental ill health and increased alcohol use may be important to include in follow-up care. In this study we found suicide risk was highest in the oldest midlife women, in contrast to general population suicide statistics where the highest rates are in women aged 45–49 years or 50–54 years (varying by study year). In this study we carried out comparisons within a subgroup of women already at high risk owing to self-harm. Women in midlife who self-harm may differ from other women in midlife, potentially with more complex needs and longer history of mental ill health accounting for the difference in distribution of suicide risk across midlife.

Within-group differences identified in this work indicate that women in midlife are not homogenous, and broad age comparisons may obscure important differences. A number of within-midlife differences occurred between the 45–49 and 50–54 year age bands, consistent with evidence that menopause typically occurs in women in their early 50s, and is accompanied by a number of changes that can have a detrimental impact on both physical and mental health.^18^ Furthermore, while physical health problems increased across midlife age bands, mental health problems were more variable, with women aged 50–54 most often reporting this as a precipitant of self-harm (33%).

Some characteristics related to self-harm in women in midlife may be an artifact of experiences that naturally tend to increase age, such as physical health problems and bereavement.^19,20^ However, the presence of these factors alongside additional indicators of risk, such as increased alcohol use or financial concerns, may make women in midlife more vulnerable to self-harm, suicide and mental health problems more generally.^4,5,7,21^

Problems with alcohol were common in women across midlife, with no significant difference identified between midlife age bands. This is consistent with literature from Western countries that alcohol consumption has increased in women in general, and in women in midlife in particular.^7,22,23^ In a systematic review of qualitative studies, alcohol use in women in midlife was associated with many factors, including respite from stressful circumstances and a reduction of familial and economic responsibilities (e.g. no longer caring for children, approaching retirement, etc.) that often occur in midlife.^24^

### Limitations

This study used complete cohort data from three cities in England and may not be generalisable to other settings. However, the sociodemographic and economic diversity of the local populations at each site is likely to be a good general representation of hospital presenting self-harm in other urban areas. Most people who self-harm do not contact services, and those who present to the emergency department may differ systematically from those in the community. Survey work suggests self-harm in the community has increased over time, whereas hospital presentations for self-harm have been relatively stable in recent years.^25^ While this is a recognised limitation of self-harm work based on patient records and service contact, presentation to services provides an important opportunity to provide quality care and implement self-harm-specific interventions. The study was restricted to operationalised information collected via medical records and clinical staff, and some potentially important situational and social factors could not be included (e.g. domestic violence, caring responsibilities, changes in physical appearance, loss of job roles and approaching retirement). It was not possible to assess whether physical and/or mental health problems might be associated with menopausal changes in women in midlife and interpretation therefore draws on previous research.^11,26^ Given the known impact of the menopause on mental health and suicidality, it is likely to play some role in self-harm in midlife, but more research is needed.^11,12^ It was not possible to adjust for mental health diagnoses or other indicators, such as receipt of psychotropic medication, because of a lack of information in hospital records and emergency department psychosocial assessments from which our data are derived. Similarly, we were unable to adjust for suicidal intent as this is not recorded on the Multicentre Study database.

### Implications

Midlife is a time of transition with specific challenges and changing care needs for women who self-harm. While midlife women more often received a specialist psychosocial assessment compared to younger women, increased risk of alcohol involvement and suicide mortality is of concern. The results suggest women in midlife who self-harm have specific social stressors that could be addressed to reduce risk, such as changing family relationships, unstable employment and problems with finances, alongside physical and mental health problems, and increased alcohol use. Awareness of such potential vulnerabilities may help inform clinicians in risk formulation, care and safety planning. Women in midlife are not a homogenous group, with both risks and care/support needs changing across the midlife period. Further research on self-harm and suicide with a focus specifically on women may also help to reduce the existing gender health gap.

## Supporting information

Clements et al. supplementary material 1 Clements et al. supplementary material

Clements et al. supplementary material 2 Clements et al. supplementary material

## Data Availability

Individual patient-level is not available because of confidentiality concerns and data-sharing agreements currently in place. The study protocol, statistical analysis plan and analytic code are available on request from the corresponding author.
