# Supplementary material for: Self-harm in women in midlife: rates, precipitating problems and outcomes following hospital presentations in the multicentre study of self-harm in England
Source: Br J Psychiatry. 2025 Jul;227(1):456–62. doi: 10.1192/bjp.2024.215 (PMC12278048; doi:10.1192/bjp.2024.215)
Supplement: Clements et al. supplementary material 1 — Clements et al. supplementary material [file S0007125024002150sup001.docx]

**Figure 1.** Flow chart describing cases included/excluded from statistical analyses.

**Multicentre Study database 2003-2016**

*Women aged 18-years and over:*

Emergency department presentations n=**51,036**

Made by n=**25,610** individuals

**Within-group midlife-comparison, individual-level analysis** (total n=6,441)

*Characteristics & repetition analysis*

40-44 years n=**2,558** assessed n=**1,761** (69%)

45-49 years n=**1,908** assessed n=**1,327** (70%)

50-54 years n=**1,285** assessed n=**904** (70%)

55-59 years n=**690** assessed n=**502** (73%)

*Trends over time* (annual index episodes)

40-44 years n=**2260**

45-49 years n=**1786**

50-54 years n=**1195**

55-59 years n=**559**

*Mortality follow-up to 2019* (% of age-band n)

Data available for:

40-44 n=**2,432** (95%)

45-49 n=**1,823** (96%)

50-54 n=**1,229** (96%)

55-59 n=**663** (96%)

**Cases included in between-group comparative individual-level analysis**

*Characteristics & repetition analysis*

Women in midlife 40-59 years: n=**6,441**

- assessed cases n=**4,494** (69.8%)

Younger women 25-39 years: n=**8,850**

- assessed cases n=**5,966** (67.4%)

*Trends over time* (annual index episodes)

Midlife yearly index presentations n=**5800**

Younger women yearly index presentations n=**7742**

*Mortality follow-up to 2019* (% of group n)

Data available for:

Midlife n=**6,147** (95%)

Younger n=**8,133** (92%)
