# Supplementary material for: Self-harm in women in midlife: rates, precipitating problems and outcomes following hospital presentations in the multicentre study of self-harm in England
Source: Br J Psychiatry. 2025 Jul;227(1):456–62. doi: 10.1192/bjp.2024.215 (PMC12278048; doi:10.1192/bjp.2024.215)
Supplement: Clements et al. supplementary material 2 — Clements et al. supplementary material [file S0007125024002150sup002.docx]

**Supplementary Table S1 overall comparisons**

| **All cases included in analysis** | | | |
| --- | --- | --- | --- |
| **Variable** | **Women aged 40-59**  **n=6,441 (%)** | **Women aged 25-39**  **n=8,850 (%)** | **X2 (d.f) P value** |
| *Primary self-harm method* | | | |
| Self-poisoning | 5,468 (84.9) | 7,059 (79.8) | 66.8 (2)p<0.001* |
| Self-injury | 799 (12.4) | 1,451 (16.4) |  |
| Self-poisoning & Self-injury | 174 (2.7) | 340 (3.8) |  |
| *Alcohol involved* | 3,091 (64.3) | 3,796 (59.5) | 27.3 (1) p<0.001* |
| *Assessed by psych services* | 3,999 (62.1) | 5,118 (57.8) | 27.9 (1) p<0.001 |
| **Assessed cases included in analysis** | | | |
|  | N=4,494 | N=5,966 |  |
| *Employment status* | | | |
| Unemployed | 1,269 (31.8) | 1,690 (31.3) | 9.2 (2) p=0.010 |
| Employed | 1,616 (40.6) | 2,341 (43.4) |  |
| Other (including student) | 1,100 (27.6) | 1,365 (25.3) |  |
| *Ethnicity* | | | |
| White | 3,873 (93.5) | 4,746 (85.3) | 161.7 (3) p<0.001* |
| Black | 77 (1.9) | 206 (3.7) |  |
| South Asian | 104 (2.5) | 358 (6.4) |  |
| Other non-white | 90 (2.2) | 256 (4.6) |  |
| *History of self-harm* | 2,209 (55.0) | 2,991 (55.9) | 0.7 (1) p=0.415 |
| *Current psychiatric care* | 975 (23.7) | 1,105 (20.4) | 14.6 (1) p<0.001* |
| *Problems reported as precipitants of self-harm* | | | |
| Relationship with partner | 1,806 (41.5) | 2,851 (49.9) | 69.1 (1) p<0.001* |
| Relationship with family | 1,069 (24.6) | 1,383 (24.2) | 0.2 (1) p=0.672 |
| Relationship with others | 570 (13.1) | 622 (10.9) | 11.6 (1) p=0.001* |
| Employment/Study | 676 (15.5) | 885 (15.5) | 0.0 (1) p=0.954 |
| Financial | 814 (18.7) | 863 (15.1) | 23.1 (1) p<0.001* |
| Housing | 565 (13.0) | 716 (12.5) | 0.5 (1) p=0.491 |
| Legal | 171 (3.9) | 265 (4.6) | 3.0 (1) p=0.085 |
| Alcohol | 937(25.2) | 1,018 (21.5) | 16.6 (1) p<0.001* |
| Drugs | 125 (3.2) | 311 (6.3) | 43.9 (1) p<0.001* |
| Health | 689 (15.9) | 529 (9.3) | 100.6 (1) p<0.001* |
| Mental health | 1,318 (30.3) | 1,569 (27.46) | 9.6 (1) p<0.001* |
| Bereavement | 596 (13.9) | 527 (9.4) | 50.8 (1) p<0.001* |
| Abuse | 469 (10.8) | 732 (12.9) | 9.8 (1) p=0.001* |
